# Suppressing Spurious Causal Chains in Alarm-Triggered Alarm Phenomena via Liang–Kleeman Information Flow–Max-Min Hill-Climbing Graphs

**DOI:** 10.3390/s26206573

**Published:** 2026-10-16

**Authors:** Xiaolu Li, Shuaixing He, Jie Liu, Zhi Chen, Xiaohua Yang, Zhuoran Xu

**Affiliations:** 1 School of Computer Science, University of South China, Hengyang 421001, China; 2 Hunan Engineering Research Center of Software Evaluation and Testing for Intellectual Equipment, Hengyang 421001, China; 3 Hangzhou Xiangting Technology Co., Ltd., Hangzhou 311100, China; 4 National Key Laboratory of Nuclear Reactor Technology, Nuclear Power Institute of China, Chengdu 610213, China

**Keywords:** spurious alarm, alarm flooding, critical alarm, causal propagation chains

## Abstract

Due to coupling in industrial control structures, alarm variables may exhibit interconnected variations, leading to the “alarm-triggered alarm” phenomenon, which can contribute to alarm flooding. Causal inference methods can characterize dependencies among alarm variables and help identify potential propagation paths for alarm configuration optimization. However, strong autocorrelation, nonlinear dynamics, and time-delay effects in complex industrial processes may lead conventional causal inference methods to identify spurious causal relationships, thereby reducing the reliability of the resulting causal graphs. To address this issue, we propose a Liang–Kleeman information flow–Max-Min Hill-Climbing (IF-MMHC) causal graph framework that combines physics-informed information flow analysis with constrained score-based structure learning. Specifically, Liang–Kleeman information flow (LKIF) separates endogenous evolution from external driving effects and provides physically informed constraints for identifying candidate causal relationships, thereby reducing spurious causal effects associated with temporal dependence. The resulting candidate graph is then optimized using the Max-Min Hill-Climbing (MMHC) algorithm through constraint checking and score-based structural search, further removing redundant and indirect edges and improving the structural parsimony of the causal graph. Experiments on multiple fault scenarios of the Tennessee Eastman Process demonstrate the effectiveness of IF-MMHC in identifying key alarms and causal propagation relationships while reducing redundant causal connections. The results provide evidence for the applicability of the proposed framework to industrial alarm analysis and configuration optimization, while broader validation across different industrial processes remains necessary.

## 1. Introduction

An alarm system is a vital component for status monitoring within industrial control systems. In complex industrial manufacturing processes, status variables can typically be categorized into three distinct hierarchical levels: fault variables, which characterize the intrinsic status of system anomalies or faults and reflect the root cause of the system’s deviation from normal operation, are often not directly observable or are difficult to measure accurately via sensors in practical industrial scenarios; monitored variables refer to the process status variables that can be directly collected or acquired by sensors or the system itself; and alarm variables denote the status variables triggered based on monitored variables through threshold determination or logical rules, which are utilized to indicate whether a system anomaly has occurred and to initiate alarm signals.

Because fault variables are difficult to observe directly, industrial systems typically rely on monitored variables for indirect characterization to implement alarming. However, indiscriminately configuring all fault-related monitored variables as alarm variables can easily lead to information redundancy within the alarm system, resulting in alarm floods [1] and consequently increasing the risks of misjudgment and human error when hazards occur. Therefore, it is essential to screen and select an optimal subset from the candidate set of related monitored variables to serve as alarm variables.

Due to the temporal differences or mutual coupling in the responses of multiple monitored variables to the same fault variable, their corresponding alarm variables exhibit sequential or linked changes, which subsequently causes certain alarm variables to be indirectly driven by other alarm variables in addition to responding to the fault. This “alarm-triggers-alarm” phenomenon induced by the control structure is, in essence, a causal chain transmitted from the coupling among status variables to the alarm variables; that is, downstream alarms are frequently driven indirectly by upstream alarms rather than directly responding to the fault, thereby generating false alarms. Therefore, identifying and parsing the causal chains transmitted from status variable coupling to alarm variables constitutes a critical approach to achieving alarm configuration optimization.

Traditional alarm analysis methods often rely on static rules, empirical configurations, or predefined process knowledge. These approaches can be difficult to apply to complex industrial processes, where alarm variables may interact dynamically and their responses may propagate through coupled process structures. Causal inference provides a useful framework for characterizing directed dependencies among alarm variables and identifying potential propagation paths, thereby supporting alarm analysis and configuration optimization. However, the reliability of causal identification depends on the characteristics of the underlying process data. In industrial processes, strong temporal dependence, nonlinear dynamics, time delays, and process coupling may affect the ability of conventional causal inference methods to distinguish genuine causal effects from dependencies arising from the intrinsic evolution of process variables.

Several causal analysis methods have been applied to industrial alarm data. For example, transfer entropy combined with the K2 algorithm has been used to identify causal chain relationships between binary alarm variables [2]. Transfer entropy can quantify directed information transfer and is therefore useful for causal analysis; however, its estimation may be affected by strong autocorrelation in industrial process variables. In such cases, a downstream alarm may reflect the historical evolution of its own state rather than information transferred from an upstream alarm, making the interpretation of the inferred causal relationship more difficult. Other studies have combined active transfer entropy with simplified multilayer Bayesian networks to identify latent causal structures in alarm sequences [3]. These approaches improve the ability to characterize dynamic alarm relationships, but the temporal dependence and structural coupling inherent in industrial processes remain important challenges.

Knowledge-driven and data-driven approaches have also been combined for alarm causal analysis. For instance, knowledge extracted from Hazard and Operability Analysis (HAZOP) has been integrated with an Autoregressive Integrated Moving Average (ARIMA) model and Granger causality analysis to trace causal relationships [4]. In this context, HAZOP provides process knowledge concerning deviations, their potential causes, and consequences, while ARIMA is used to characterize the temporal behavior of process variables. Granger causality is then applied to examine directional temporal relationships among the variables. These studies illustrate how process knowledge and time-series modeling can be combined to support alarm causal analysis. However, ARIMA itself is primarily a time-series modeling approach rather than a method specifically designed to identify alarm causality. Therefore, additional causal analysis is required when the objective is to distinguish direct causal relationships from temporal dependencies in coupled industrial processes.

A major challenge arises from the pronounced time delays and feedback mechanisms commonly present in industrial processes [5]. The current state of an alarm variable may be strongly related to its historical states, while its observed temporal relationship with another variable may also result from external driving effects. Liang–Kleeman information flow provides a quantitative framework for analyzing directed information transfer based on the evolution of stochastic dynamical systems [6]. By decomposing the entropy rate evolution of a variable into contributions associated with its own dynamics and external driving variables, LKIF provides a means of distinguishing externally transferred information from the variable’s intrinsic evolution. This property is particularly useful for industrial process data with strong temporal dependence and time-delay effects, where conventional dependency measures may not clearly distinguish the source of the observed relationship. Therefore, LKIF can provide physically informed causal constraints for subsequent causal structure identification.

Another challenge is that multiple monitored variables may respond to the same fault with different time delays or through coupled process dynamics [7]. As a result, the inferred causal structure may contain multiple candidate relationships, including direct and indirect connections. A large number of candidate edges increases the structural search space and may make it difficult to obtain a concise causal graph. The Max-Min Hill-Climbing (MMHC) algorithm provides a hybrid structure-learning strategy that combines constraint-based candidate selection with score-based structure optimization [8]. The constraint-based stage identifies a set of candidate parent–child relationships, while the subsequent score-based search optimizes the graph structure by evaluating alternative edge configurations. MMHC is therefore suitable for further refining a causal graph after an initial set of physically informed candidate relationships has been obtained.

Based on these considerations, this study integrates Liang–Kleeman information flow with MMHC to construct a constrained causal graph for industrial alarm analysis. The two components serve complementary roles in the proposed framework. First, LKIF is used to quantify directed information flow between monitored variables. Information flow relationships satisfying the predefined significance criterion are then used as physics-informed prior constraints to construct candidate parent–child sets and restrict the subsequent structure-learning search space. Second, MMHC performs constraint-based candidate selection followed by score-based structural optimization within the restricted search space. Through this combination, LKIF provides the causal constraints derived from the directional information flow analysis, whereas MMHC optimizes the remaining graph structure and removes candidate relationships that provide limited improvement to the overall graph score. The resulting framework therefore combines physically informed causal characterization with constrained data-driven structure learning.

The proposed workflow first performs data cleaning, binary discretization, and sliding-window smoothing on the monitored variables, followed by alarm subset selection with reference to expert knowledge. LKIF is then calculated for pairs of monitored variables, and statistically significant information flow relationships are used to construct an initial directed graph. After cycle removal processing, the resulting information flow structure is provided to MMHC as a prior constraint. Candidate parent–child sets are constructed from the retained information flow relationships after the predefined information flow filtering and cycle removal procedures. The resulting CPC sets define the candidate search space for the subsequent MMHC structure-learning procedure rather than directly determining the final causal edges, and greedy hill-climbing search is subsequently performed to optimize the graph structure. This procedure aims to retain the causal relationships that provide meaningful structural information while reducing unnecessary connections and producing a sparse and physically interpretable causal graph.

The main contributions of this study are as follows. First, an integrated LKIF-MMHC framework is developed for industrial alarm causal analysis, in which directional information flow analysis is used to constrain subsequent causal structure learning. Second, the proposed framework combines physics-informed causal characterization with constrained score-based graph optimization, allowing the causal search space to be reduced before structural optimization. Third, the effectiveness of the proposed approach is evaluated under multiple fault scenarios of the Tennessee Eastman Process, with comparisons to representative causal analysis approaches including transfer entropy and Granger causality. The experimental results provide evidence for the applicability of the proposed framework to alarm causal analysis and configuration optimization, while validation on additional industrial processes remains an important direction for future work.

The remainder of this paper is structured as follows: Section 2 reviews related work on alarm configuration optimization; Section 3 presents the theories and methodological workflow of LKIF and MMHC; Section 4 evaluates the proposed method using the Tennessee Eastman Process simulation; Section 5 compares the proposed method with approaches such as transfer entropy and Granger causality in identifying critical alarms; and Section 6 summarizes the main findings and discusses future research directions.

## 2. Related Work

To optimize alarm configuration and improve the management of alarm flooding, existing studies have explored a variety of approaches. These methods can be broadly grouped into three categories: correlation-based analysis, sequence pattern mining, and causal inference. In addition, Bayesian networks and, more recently, deep learning-based approaches have been investigated for alarm root-cause analysis and fault diagnosis. These methods provide complementary perspectives for characterizing relationships among alarm variables, but they differ in their ability to represent temporal dependencies, causal mechanisms, and process dynamics.

In correlation-based analysis, statistical relationships among alarm variables are quantified and used to support alarm flooding identification and configuration optimization. For example, one study proposed an alarm flooding similarity analysis method that combines global vectors with a modified move–split–merge (MSM) distance. The method compares encoded alarm sequences with different lengths to identify root alarms associated with alarm flooding and support configuration optimization [9]. Another study extracted statistical features from historical alarm and event (A&E) logs to identify correlations among alarms, providing information for alarm configuration and alarm suppression strategies [7]. To account for temporal offsets, a time-delay block matching similarity method was also proposed to estimate delayed associations between alarm variables using maximum block coefficients, thereby improving the temporal sensitivity of configuration optimization [10]. These studies demonstrate that correlation-based analysis can effectively quantify statistical associations and identify related alarm variables. However, statistical association does not necessarily indicate a directional causal relationship. In particular, when alarm triggering involves time delays or coupled process dynamics, two alarms may exhibit strong statistical similarity without one directly causing the other. Therefore, although correlation-based methods are useful for identifying related alarms and supporting configuration optimization, their ability to reconstruct abnormal propagation mechanisms remains limited when explicit temporal and causal information is required.

To incorporate the ordering and co-occurrence of alarm events, sequence pattern mining methods have been applied to identify frequent alarm sequences, detect redundant alarms, and support dynamic configuration optimization. For example, an association rule-mining algorithm was developed to predict alarm flooding events and their response times, providing information for dynamic alarm configuration [11]. Another study employed sequence pattern mining to identify temporal patterns among alarms and represented the resulting patterns using statistical features to characterize interactions among process variables [12]. In addition, an FP-Growth method combined with the J-Measure was proposed to identify first-occurring alarms and extract alarm rules from historical sequences, which were then used to identify potentially redundant alarms [13]. Compared with purely static correlation analysis, sequence pattern mining explicitly considers event order and therefore provides additional information about the temporal organization of alarm sequences. However, the patterns identified by these methods primarily describe recurring co-occurrence relationships rather than causal mechanisms. Small variations in alarm occurrence times may also fragment otherwise similar patterns, particularly in processes with variable response delays. Furthermore, noise, operating condition changes, and complex process coupling may affect the stability of the extracted patterns. Consequently, sequence pattern mining is useful for discovering temporal alarm patterns, but additional causal analysis is required when the objective is to determine why one alarm propagates to another.

Causal inference methods aim to move beyond statistical association and sequence co-occurrence by identifying directional relationships among alarm variables. This provides a basis for analyzing potential triggering mechanisms and reconstructing alarm propagation paths. For example, one study combined transfer entropy with an improved conditional mutual information measure to identify causal chains in multi-valued alarm sequences [14]. This approach uses information-theoretic measures to characterize directional dependencies while incorporating conditional information to reduce the influence of irrelevant variables. Another study employed active dynamic transfer entropy to construct propagation networks from alarm time series and extract causal propagation chains during alarm evolution [15]. In addition, Granger causality-based approaches have been used to identify temporal precedence relationships between alarm variables and support alarm configuration optimization [16]. These studies demonstrate the value of causal inference for identifying directional alarm relationships and interpreting potential propagation mechanisms. Nevertheless, industrial alarm data have characteristics that can affect causal identification. For example, transfer entropy-based measures may be influenced by strong temporal dependence in process variables, making it difficult to distinguish information transferred from another variable from dependencies associated with the variable’s own evolution. Granger causality provides a useful test of whether past observations of one time series improve the prediction of another, but its conventional formulation is prediction-oriented and does not directly provide a quantitative measure of directional information flow strength. Consequently, when reciprocal temporal dependencies exist, the relative contributions of the two directions may be difficult to characterize solely from Granger causality results. These limitations motivate the use of causal measures that explicitly account for the dynamic evolution of industrial process variables.

Bayesian networks (BNs) and related causal graphical models are also widely used for alarm root-cause analysis because they provide an explicit graphical representation of conditional dependencies among process variables [17]. Conventional BN structure learning can identify probabilistic dependencies from alarm sequences through conditional-independence tests or scoring functions. This provides a structured representation of alarm relationships and can facilitate root-cause reasoning. However, when applied directly to discretized alarm sequences, conventional BN structure learning does not explicitly distinguish a variable’s intrinsic temporal evolution from information transmitted by other variables. This limitation becomes important for industrial processes with strong autocorrelation and time-delay effects.

More recently, deep learning-based methods have been introduced into industrial fault diagnosis, process monitoring, and causal or relational analysis. For example, graph neural networks and attention mechanisms have been used to model complex relationships among process variables and improve fault diagnosis in industrial processes [18]. Graph attention-based Bayesian networks have also been investigated for fault root-cause analysis by combining learned representations with prior structural information [17]. In addition, knowledge data-driven approaches based on temporal knowledge graphs and supervised contrastive learning have been developed for monitoring complex industrial processes [19]. These studies demonstrate the potential of deep learning to represent high-dimensional, nonlinear, and structurally complex dependencies in industrial systems. However, deep learning-based approaches generally rely on learned representations and model architectures, and their causal interpretation may be less explicit than that of methods formulated directly in terms of causal information transfer. Moreover, they do not necessarily distinguish a variable’s endogenous evolution from information transmitted by external variables, which is an important consideration for the alarm causal analysis problem addressed in this study. Therefore, deep learning approaches provide a complementary data-driven perspective, while physically interpretable causal measures remain valuable when causal mechanisms and alarm propagation paths need to be explicitly analyzed.

Accordingly, this study proposes an information flow–Max-Min Hill-Climbing (IF-MMHC) framework for industrial alarm configuration analysis. Liang–Kleeman information flow (LKIF) is used to quantify directional information transfer and provide physically informed constraints for identifying candidate causal relationships. The resulting candidate relationships are then supplied to the Max-Min Hill-Climbing (MMHC) algorithm, which performs constrained structure learning and score-based optimization to refine the causal graph. In this way, LKIF and MMHC serve complementary roles: LKIF provides the directional and physically informed constraints, while MMHC optimizes the graph structure within the constrained search space. The proposed framework aims to identify meaningful causal relationships and propagation pathways while obtaining a more concise and physically interpretable causal graph. The effectiveness of the framework is evaluated using multiple fault scenarios of the Tennessee Eastman Process, with comparisons against representative causal analysis methods. Validation on additional industrial processes and broader operating conditions remains an important direction for future work.

## 3. Suppressing Spurious Causal Chains in Alarm-Triggered Alarm Phenomena via IF-MMHC Graphs

**Figure 1:**
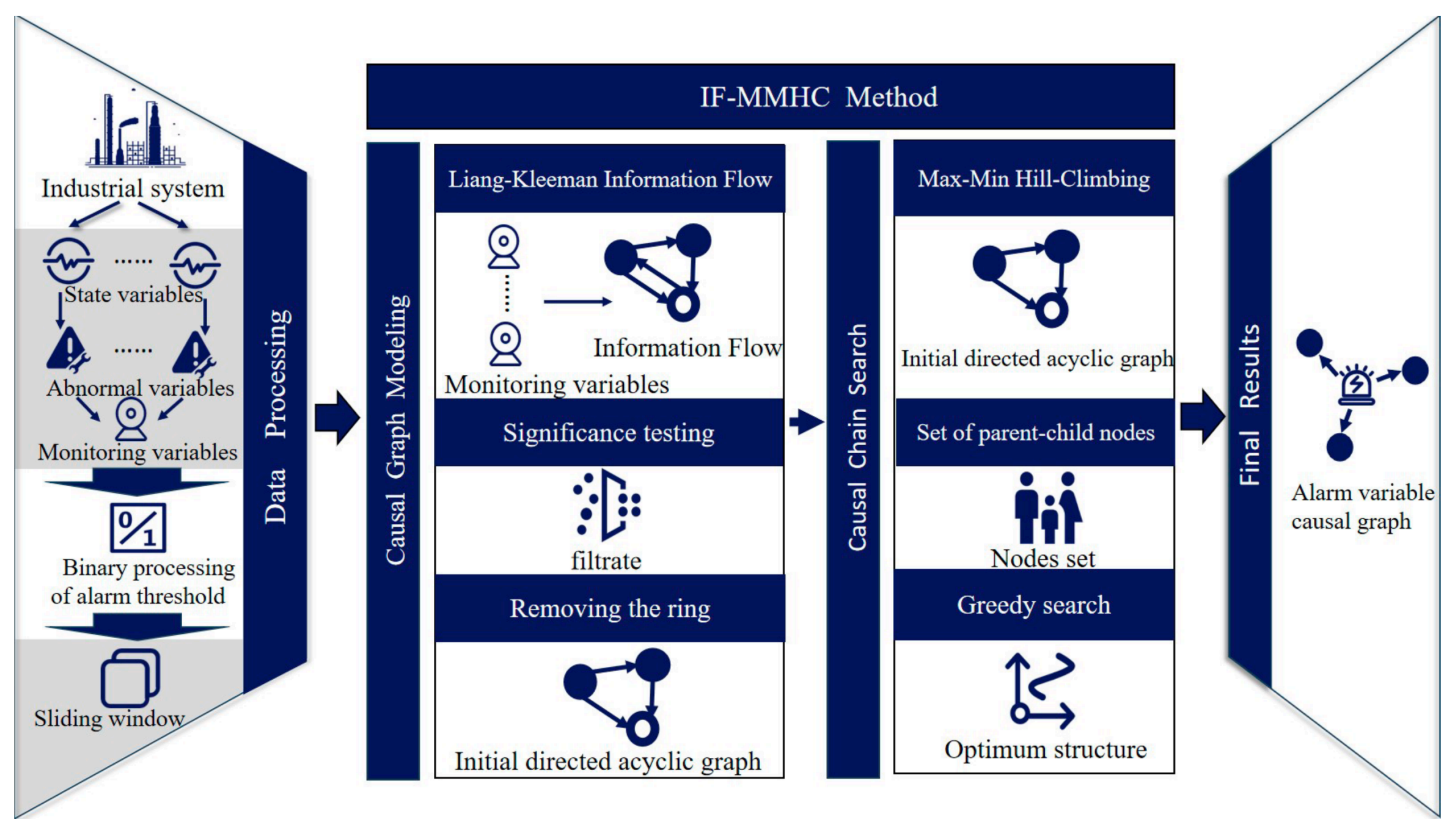
Method flowchart.

This section formalizes the IF-MMHC framework outlined in Figure 1 and depicts the three constituent stages—data preprocessing, LKIF-based causal graph modeling, and MMHC-based causal chain search—and Section 3.1, Section 3.2 and Section 3.3 detail each stage in turn, following the block structure of Figure 1.

Because fault variables may not be directly observable, monitored process variables are used as their observable indicators. The raw sensor readings are first cleaned and discretized into binary alarm states using a sliding-window threshold rule. Expert knowledge and historical operating information are then used to select monitored variables that are relevant to the investigated fault scenarios, rather than to determine unobservable fault variables. LKIF is subsequently applied to the selected variables to quantify directional information flow. After significance filtering and cycle removal, the retained relationships form an initial directed acyclic graph. This graph is then used to construct candidate parent–child sets for MMHC, which performs a constrained greedy hill-climbing search to obtain the final causal graph for alarm propagation analysis.

### 3.1. Data Processing

Before causal analysis, the continuous process measurements were first converted into alarm events according to the predefined upper and lower alarm thresholds *H_a_* and *L_a_*, respectively. These thresholds were determined using the 3σ criterion. A measurement exceeding *H_a_* or falling below *L_a_* was assigned an alarm state of 1, while the remaining observations were assigned an alarm state of 0. The resulting alarm events were therefore represented as binary alarm-state sequences, where 1 denotes an alarm state and 0 denotes a non-alarm state.

To reduce short-term fluctuations and alarm chattering, a moving-average operation with a window length of 10 samples was applied to the binary alarm sequences, as shown in Table 1. Since the moving-average operation produces values between 0 and 1, the averaged alarm state was converted back to a binary representation using a threshold of 0.5; values greater than 0.5 were assigned to 1, whereas values less than or equal to 0.5 were assigned to 0. A step size of 2 samples was used for moving-window processing [20]. The resulting binary alarm-state sequences were then used as the input to the subsequent sliding-window IF analysis. The resulting binary alarm-state sequences are discrete-time data and are used directly as the input to the numerical LKIF estimator. The moving-average and re-binarization procedures are intended to reduce short-term fluctuations and alarm chattering and are not intended to analytically remove autocorrelation.

The experimental process data contain measurement and process-related noise. The moving-average preprocessing described above is applied to reduce short-term fluctuations and alarm chattering in the alarm-state sequences before IF estimation. No additional white-noise removal or pre-whitening procedure is applied. The sliding-window strategy is used to obtain local estimates under potentially time-varying alarm relationships, rather than assuming global stationarity over the entire observation period.

### 3.2. Causal Graph Modeling

To more accurately characterize the dependency structure among alarm variables, the monitored variables are first represented as binary alarm-state sequences according to the alarm-state definition described in Section 3.1. Expert knowledge and historical operating data are then used to identify monitored variables that are relevant to the investigated fault scenarios and to construct the alarm variable subset. This step is limited to variable selection and does not manually determine causal relationships or assign causal strengths.

Based on the selected monitored variables, Liang–Kleeman information flow (LKIF) is introduced to quantify directional information transfer among the variables. LKIF provides a physically interpretable measure of directional information transfer based on the dynamical relationships among variables. In this study, the numerical estimation is performed using a linear LKIF estimator on the preprocessed binary alarm-state sequences within local sliding windows. The numerical estimator calculates the information flow quantity from covariance and finite-difference covariance quantities obtained from the observed time-series samples within each sliding window. Thus, the continuous formulation provides the theoretical basis for the LKIF measure, whereas the actual numerical estimation is performed on the discrete-time binary alarm-state sequences. Although the original continuous-time formulation is derived from the Liouville/Fokker–Planck equations, Liang–Kleeman information flow has been rigorously extended to discrete-time mappings. In particular, the principle of nil causality holds as a proven theorem for discrete systems: if the evolution of one variable does not depend on another, the corresponding information flow vanishes. Consequently, when the numerical linear estimator is applied to binary alarm-state sequences, the estimated information flow retains this causal interpretability. Detailed derivations for both continuous and discrete cases are provided in Appendix A. The underlying theoretical principle is described as follows (refer to the Appendix A for the detailed mathematical derivation):

(1) $$T_{2\to 1}=-E\left[\frac{1}{\rho_{1}}\;\frac{\partial \left(F_{1}\rho_{1}\right)}{\partial X_{1}}\right]+\;\frac{1}{2}E[\frac{1}{\rho_{1}}\;\frac{\partial^{2}\left(b_{11}^{2}+b_{12}^{2}\right)\rho_{1}}{\partial x_{1}^{2}}]$$

Here, ρ_1_ denotes the marginal probability density of *X*_1_, *B* = (*b_ij_*) represents the diffusion coefficient matrix, and E refers to the expectation with respect to the joint probability density *ρ*(*X*_1_,*X*_2_). When X_2_ exerts no influence on the evolution of X_1_, the information flow from *X*_2_ to *X*_1_ becomes zero, i.e., *T*_2→1_ = 0. This property is consistent with the zero-causality principle of the information flow framework. Therefore, LKIF provides a quantitative measure of directional information transfer that complements conventional statistical dependency measures in the analysis of alarm variable relationships.

For numerical estimation, the required statistical quantities are calculated locally within each sliding window. For each ordered pair of alarm variables, the sample covariance matrix is first calculated from the observations within the current window. The corresponding finite-difference series are then obtained using the specified time-lag parameter, and the covariance terms between the original and finite-difference series are calculated. These covariance quantities are used to estimate the coefficients required by the linear LKIF estimator and subsequently obtain the information flow from *X_j_* to *X_i_*. The window is then shifted forward according to the predefined step size, and the calculation is repeated. The time-lag parameter is set to τ in the main experiments, and its influence on the resulting causal structure is further examined in the parameter sensitivity analysis.This is detailed in Algorithm 1 below.
**Algorithm 1:** Construction of the Information Flow GraphInput: A set of binary alarm-state time series *X* = {*x*_1_, *x*_2_, …, *x_n_*}, time lag step *τ*, sliding-window length *W*, window step *s* Output: Information flow graph *G* = (*V*,*E*)1.Initialize node set *V* = {1, 2, …, *n*} and edge set *E* = *∅*2.Divide each binary alarm-state time series into overlapping windows of length *W* with step size *s*.3.For each sliding window *W* do4.  Initialize information flow set *F*^(*W*)^ = *∅.*5.   For *i* = 1 to *n* do6.    for *j* = 1 to *n*,*j* ≠ *i* do7.    Calculate the information flow from $x_{j}\;\mathrm{to}\;x_{i}\;{Τ}_{j\to i}^{\left(\tau \right)}$8.    $\mathrm{Add}\;{Τ}_{j\to i}^{\left(W\right)}$ to *F*^(*W*)^9.  End for10.End for11.  If: ${Τ}_{j\to i}^{\left(\tau \right)}$ exceeds the predefined information flow filtering threshold, then12.    *E* ← *E* ∪ {(*j*,*I*)}13.  End If14.End For15.return information flow graph *G* = (*V*,*E*)

#### 3.2.1. Construction of Causal Alarm Nodes

In the causal alarm node selection stage, candidate monitored variables are first screened based on the predefined fault scenarios and their propagation patterns, together with the process flow structure and the coupling relationships among process variables and control loops. Expert knowledge and historical operational data are used to assist this screening process and to ensure that the selected variables are relevant to the investigated fault propagation processes. Subsequently, Liang–Kleeman information flow (LKIF) is employed to quantitatively estimate the directional information flow between each pair of candidate monitored variables, thereby generating an information flow matrix. To reduce the influence of noise and incidental relationships, a predefined information flow filtering threshold is applied, and only variable pairs with information flow strengths exceeding this threshold are retained. Based on the filtered information flow relationships, the core alarm variables associated with the investigated fault sources are identified and used as the node set and causal constraints for the subsequent IF-MMHC causal graph modeling stage.

#### 3.2.2. Construction of the Causal Alarm Graph

In the construction of the causal alarm graph, Liang–Kleeman information flow (LKIF) is used to quantify the direction and strength of information transfer among the selected monitored variables. For each pair of monitored variables, the corresponding directional information flow is estimated to identify potential causal relationships. A predefined information flow filtering threshold is then applied to remove weak information flow relationships, and the retained relationships are used to construct the initial causal structure and provide directional constraints for the subsequent MMHC-based structure learning.

In this study, the LKIF significance threshold is set to 0.2. This threshold is used to retain information flow relationships with sufficient strength for subsequent causal graph construction and candidate parent–child (CPC) generation. The value of 0.2 is an experimental setting adopted in this study rather than a universal threshold for industrial processes. Its influence on the resulting causal structure and alarm identification performance is further investigated in the ablation experiments in Section 4.

After significance filtering, the retained information flow relationships are used to construct the initial causal alarm graph. The graph represents the directional information transfer relationships among the selected monitored variables and provides the basis for the subsequent CPC construction and MMHC-based structure optimization.

Specifically, let the random variables ${\left(x_{1},\ldots ,x_{n}\right)}^{T}$ follow the stochastic dynamic equation [21]:

*d_x_* = *A_x_d_t_* + *Bd_w_* (2)

where A = (a_ij_), and B is a constant matrix representing stochastic white noise, with its covariance matrix assumed as σ_ij_, for any i and j = 1, …, n, with i ≠ j. Liang-Kleeman information flow from x_j_ to x_i_ is defined as follows:

(3) $$T_{j\to i}=a_{\mathrm{ij}}\frac{\sigma_{\mathrm{ij}}}{\sigma_{\mathrm{ii}}}$$

where $a_{\mathrm{ij}}:\;F_{1}=\frac{dx_{1}}{d_{t}}$

= *f*_1_ + *a_i_*_1_*x*_1_ + *a_i_*_2_*x*_2_+…+*a_ij_x_j_*+…+*a_in_x_n_* + *b_i_*_1_*w*_1_+…+*b_in_w_n_*

Among them, σ_ij_ denotes the variance of x_i_ derived from the covariance matrix between the two-dimensional variables *x_i_* and *x_j_*, and *cov*(*x*,*y*) = *E*[(*x* − *E*(*x*))(*y* − *E*(*y*))].

Based on the above computation, once the information flow within each subgroup is obtained, causal relationships can be analyzed. The value of the information flow may be either positive or negative. The sign of the information flow value represents the direction of the estimated causal effect, whereas its absolute magnitude reflects the strength of the estimated information flow. Moreover, when the magnitude of the information flow is relatively large, it suggests a significant causal influence of *x* on *y*.

However, in industrial processes, noise interference is an unavoidable factor. For example, when $T_{x_{2}\to x_{1}}$ = 0.653 ± 0.751, $T_{x_{1}\to x_{2}}$ = 1.240 ± 0.690 is also significant, and this result alone is insufficient to conclude the existence of a unidirectional causal relationship from variable x_1_ to variable x_2_. Therefore, for such results where significance levels are close, the standardization of information flow can be introduced for further assessment, so as to identify the root-cause variables, as shown below:

(4) $$Z_{x_{2}\to x_{1}}=\left|T_{x_{2}\to x_{1}}\right|+\left|\frac{dH_{x_{1}}^{*}}{\mathrm{dt}}\right|+\left|\frac{dH_{x_{1}}^{\mathrm{noise}}}{\mathrm{dt}}\right|$$

Here, $\frac{dH_{x_{1}}^{*}}{\mathrm{dt}}$ denotes the marginal entropy of *x*_1_ itself, and $\frac{dH_{x_{1}}^{\mathrm{noise}}}{\mathrm{dt}}$ represents the marginal entropy of *x*_1_ induced by noise. Accordingly, the normalized information flow is defined as follows:

(5) $$\tau_{x_{2}\to x_{1}}=\frac{T_{x_{2}\to x_{1}}}{Z_{x_{2}\to x_{1}}}$$

Therefore, if $\tau_{x_{2}\to x_{1}}=1$, the variation in *x*_1_ is entirely attributable to *x*_2_, whereas if $\tau_{x_{2}\to x_{1}}$ approaches zero, it indicates that *x*_2_ is not a causal factor of *x*_1_.

Based on the above computational procedure, given *N* discrete time series, this stage yields an ***N×N*** matrix in which each off-diagonal element represents the Liang–Kleeman information flow value from one monitored variable to another.

#### 3.2.3. Significance Test

After obtaining the information flow strength between the monitored variables, a predefined significance threshold is applied to filter weak information flow relationships before subsequent causal structure learning [22]. In this study, the significance threshold is set to 0.2. A directional relationship (*x_i_* → *x_j_*) is retained when its information flow strength satisfies *IF_i_*_→_*_j_* ≥ *IF_threshold_*.

Meanwhile, relationships with (*IF_i_*_→*j*_ < *IF_threshold_*) are excluded from the subsequent causal structure learning. Therefore, the filtering criterion is directly determined by the magnitude of the estimated information flow, and the term “non-critical” refers to relationships whose information flow strength does not reach the predefined threshold.

The threshold is an experimental setting used in this study rather than a universal criterion for industrial processes. Its influence on the causal structure and alarm identification performance is further investigated in the ablation experiments in Section 4. The retained relationships are subsequently used as constraints for constructing the candidate parent–child sets in the MMHC-based structure learning described in Section 3.3.

Here, the significance threshold is used as a predefined filtering criterion for retaining sufficiently strong information flow relationships; it does not represent a formal hypothesis test based on a bootstrap or surrogate-data null distribution.

#### 3.2.4. Cycle Removal

After significance filtering, the retained information flow relationships may still contain directed cycles. Because the subsequent MMHC structure learning is formulated for a directed acyclic graph (DAG), these cycles need to be removed before the structure search [23]. A DAG also provides an acyclic representation of the directional dependencies among monitored variables, which is consistent with the graph structure assumed by the subsequent causal chain analysis.

To identify and remove directed cycles, a depth-first search (DFS) strategy is employed. When a cycle is detected, the edge with the smallest absolute information flow strength among the edges in that cycle is removed. Here, the absolute information flow strength (*|IF_i_*_→*j*_*|*) is used as the measure of causal strength. Thus, the edge with the minimum (*|IF|*) within a detected cycle is regarded as the weakest relationship and is removed preferentially, while the stronger information flow relationships are retained.

This process is repeated until no directed cycle remains. The resulting acyclic information flow graph is then used as the basis for the subsequent CPC construction and MMHC-based structure learning. This is detailed in Algorithm 2 below.
**Algorithm 2:** Cycle RemovalInput: Information flow graph *G* = (*V*,*E*), where edges carry weights *ω_ij_* Output: Acyclic causal graph *G′* = (*V*,*E′*)Initialize *E′* ← *E*while directed cycle *C* exists in graph *G* = (*V*,*E′*) do  Let *E_c_* ⊆ *E′* be the set of edges in cycle *C*  Select the edge with the minimum weight: (i*,j*)=argωij(i,j)∈ECmin  Remove the edge: *E′* ← *E′*\{(i^*^,j^*^)}End while

### 3.3. Causal Chain Search

Based on the acyclic information flow graph obtained after significance filtering and cycle removal, the Max–Min Hill-Climbing (MMHC) algorithm is employed for the subsequent causal structure learning [24]. In the proposed framework, the LKIF stage provides directional constraints for the candidate search space, while the subsequent MMHC stage performs score-based structure optimization within this constrained space. The numerical magnitude of LKIF is not directly incorporated into the BIC score. Thus, the two stages play different roles: LKIF provides directional candidate relationships, whereas MMHC further optimizes the network structure within the constrained search space.

The MMHC-based structure learning consists of two stages. First, the candidate parent–child (CPC) sets are constructed according to the information flow relationships retained after the threshold-based filtering described in Section 3.2.3. For each monitored variable, only variables satisfying the predefined LKIF-based filtering criterion are included in its candidate set. This restriction reduces the number of admissible structural modifications considered during the subsequent search.

Second, a greedy hill-climbing search is performed to optimize the network structure according to the Bayesian Information Criterion (BIC) score. Starting from the initial graph, admissible local operations, including edge addition and edge deletion, are evaluated within the CPC-constrained search space. Edge reversal is deliberately prohibited in the present implementation. Because the candidate relationships are already directionally constrained by the LKIF-derived information flow graph, allowing for unrestricted reversal would risk replacing a physically meaningful causal orientation with a statistically higher-scoring but causally incorrect one. In the present framework, causal correctness is regarded as having higher priority than numerical score improvement; therefore, the search is restricted to addition and deletion of edges within the directed candidate set supplied by LKIF, and does not arbitrarily reverse the retained information flow directions. An operation is accepted when it improves the BIC score, and the search continues iteratively until no admissible operation produces a further improvement. The greedy strategy is adopted because it provides an efficient local search procedure for exploring the constrained space without exhaustively enumerating all possible network structures.

Through this procedure, the final causal graph is obtained by jointly using the directional information provided by LKIF and the score-based structure optimization of MMHC. The resulting graph is then used for the subsequent causal chain and alarm identification analysis.

#### 3.3.1. Construction of Parent–Child Node Sets Based on Information Flow

Unlike the traditional MMHC approach, which constructs the candidate parent–child (CPC) sets using conditional independence tests, this study uses the significant information flow relationships obtained by LKIF to construct the CPC sets. The significance threshold used here is the same LKIF threshold defined in Section 3.2.3, i.e., (*IF_threshold_* = 0.2). Thus, no additional threshold is introduced at this stage.

For a target monitored variable (*X_j_*), all monitored variables (*X_i_*) satisfying the directional information flow criterion ${\mathrm{IF}}_{X_{i}\to X_{j}}\;\geq \;{\mathrm{IF}}_{\mathrm{threshold}}$ are included in the CPC set of (*X_j_*), which can be expressed as

(6) $$\mathrm{CPC}=\left\{X_{i}\;\right|\left|{\mathrm{IF}}_{X_{i}\to X_{j}}\right|\;\geq \;{\mathrm{IF}}_{\mathrm{threshold}}\},$$

where X_i_ denotes a candidate variable and X_j_ is the target variable, and IF_threshold_ denotes the significance threshold.

This procedure differs from the conventional MMHC CPC construction in that candidate relationships are determined using LKIF-based information flow relationships rather than conditional independence tests. The structure-learning data consist of the preprocessed binary alarm-state sequences described in Section 3.2. The CPC sets define the admissible candidate relationships rather than fixing the final edge set. Therefore, an LKIF-retained relationship is not necessarily retained in the final causal graph, and candidate edges can still be removed during the subsequent structure search if their deletion improves the BIC score. Specifically, the Bayesian Information Criterion (BIC) is used to compare admissible graph structures within the LKIF-constrained search space. The numerical magnitude of LKIF is used only for threshold-based filtering to determine the admissible candidate relationships and is not directly incorporated as a weighting term in the subsequent BIC score. Thus, LKIF provides the directional candidate constraints, while the BIC-based MMHC search determines the final graph structure. Within the LKIF-constrained candidate search space, MMHC evaluates admissible candidate graph structures using the BIC score and iteratively refines the graph structure. Therefore, LKIF-filtered relationships are treated as candidate constraints rather than automatically accepted as final causal edges.

The time-lag parameter used in LKIF estimation is not represented as an explicit time-indexed node in the MMHC stage. Instead, it is used to estimate directional information flow from the local alarm-state sequences, and the resulting directional relationships are used to constrain the candidate graph. Therefore, the MMHC search is performed on the monitored alarm variables rather than on explicitly time-lagged edges such as *X*(*t* − *τ*) → *Y*(*t*). This is detailed in Algorithm 3 below.
**Algorithm 3:** Construction of Candidate Parents and Children Set Input: Multivariate time-series dataset *X* = {*x*_1_, *x*_2_, …, *x_n_*}, time lag τ, significance threshold *θ* Output: Candidate parent–child sets ${\left\{{\mathrm{CPC}}_{i}\right\}}_{i=1}^{n}$
 For *i* = 1 do  Initialize *CPC_i_* ← ∅    For *j* = 1 to *n*,*j* ≠ *i*,do    Calculate information flow ${Τ}_{j\to i}^{\left(\tau \right)}=\mathrm{LKIF}(x_{j}(t-\tau ),x_{i}(t))$     If ${Τ}_{j\to i}^{\left(\tau \right)}\geq \;\theta \;\mathrm{then}$          ${\mathrm{CPC}}_{i}\leftarrow {\mathrm{CPC}}_{i}⋃\;\{x_{j}\}$     End if    End for End for$\mathrm{Return}\;{\left\{{\mathrm{CPC}}_{i}\right\}}_{i=1}^{n}$

#### 3.3.2. Greedy Hill-Climbing Search

In the score-based search phase, a greedy hill-climbing strategy is employed to optimize the causal graph within the CPC-constrained search space. Starting from the initial graph, admissible local operations, including edge addition and edge deletion, are evaluated within the CPC-constrained search space. Edge reversal is not allowed in the present implementation because the candidate relationships are directionally constrained by the LKIF-derived information flow graph. An operation is accepted when it improves the BIC score, and the search continues iteratively until no admissible operation produces a further improvement.

Each candidate graph is evaluated using the Bayesian Information Criterion (BIC), which provides a trade-off between the goodness of fit of the graph and its structural complexity. The BIC score is used to compare candidate structures, with a higher score indicating a preferable balance between data fitting and model complexity. At each iteration, the admissible operation that provides the greatest improvement in the BIC score is selected. The corresponding graph is then used as the starting point for the next iteration. The search terminates when no admissible operation can further improve the BIC score, and the resulting graph is taken as the final causal graph.This is detailed in Algorithm 4 below.
**Algorithm 4:** Hill-Climbing SearchInput: Initial causal graph *G*_0_ = (*V*,*E*), candidate sets {*CPC*}*_i_*, score function Score(·) Output: Final causal graph G^*^Initialize *G* ← *G*_0_RepeatGenerate a set of candidate graphs G, including:For any edge *i* → *j*, add edge *i* → *j* if and only if *x_i_* ∈ *CPC_j_* and the information flow direction constraint is satisfiedDelete an existing edge *i* → *j* when the resulting graph remains admissible.For all *G″*∈ G, calculate Score(*G″*)Select the optimal graph G^*^ = *argmaxScore*(*G″*)If *Score*(G^*^) *> Score*(*G*) then *G* ← G^*^ else stopuntil convergenceReturn G^*^

Because the search is restricted by the CPC sets constructed from the retained information flow relationships, the number of candidate structural modifications is reduced compared with an unrestricted search over all possible edges. The greedy hill-climbing procedure therefore provides an efficient way to optimize the graph structure within the LKIF-constrained search space.

### 3.4. Evaluation Metrics for Alarm Configuration Optimization

To comprehensively evaluate the performance of different methods in identifying key alarms and alarm propagation paths, this study adopts the AC@k and Avg@k metrics proposed in existing work [25]. These ranking-based metrics are used to evaluate whether the identified alarms contain the known fault-source variables and how early these variables appear in the ranked results.

The fault scenarios evaluated in this study are listed in Table 2, including [actual IDV IDs]. For each scenario, the corresponding fault-source variable(s) specified by the predefined TEP fault configuration are used as the reference root causes for AC@k and Avg@k evaluation.

For each fault scenario, the true root causes refer to the fault-source variables specified by the corresponding Tennessee Eastman Process (TEP) fault scenario. These variables are determined from the predefined fault configuration and are treated as the reference root-cause variables for evaluation. They are not inferred from the proposed method. Let $V_{a}^{\mathrm{rc}}$ denote the set of true root-cause variables for fault case (a).

The AC@k metric evaluates whether the top-(k) ranked results contain the true root causes. It is defined as follows:

(7) $$\mathrm{AC}@k=\frac{1}{\left|A\right|}\sum_{a\in A}\frac{\sum_{i<k}R^{a}\left[i\right]\in V_{a}^{\mathrm{rc}}}{\min(k,\left|V_{a}^{\mathrm{rc}}\right|)}$$

where A denotes the set of all fault samples, *R^a^*[*i*] is the i-th ranked result for fault case a, $V_{a}^{\mathrm{rc}}$ is the set of true root causes for fault case a, and $\sum_{i<k}R^{a}\left[i\right]\in V_{a}^{\mathrm{rc}}$ is an indicator function that returns 1 if *R^a^*[*i*] is a true root cause, and 0 otherwise. The term $\min(k,\left|V_{a}^{\mathrm{rc}}\right|)$ ensures normalization to prevent excessive penalization.

The Avg@k metric is calculated as follows:

(8) $$\mathrm{Avg}@k=\frac{1}{k}\sum_{j=1}^{k}\mathrm{AC}@j$$

It provides an aggregated evaluation of the ranking performance from the first result to the (k)-th result.

## 4. Case Study

Based on the preceding process analysis, the Tennessee Eastman (TE) process simulation platform is selected as the experimental benchmark to evaluate the proposed alarm configuration optimization method. Fault datasets are generated using the TE process simulation, and the monitored variables associated with the investigated fault scenarios are selected according to expert knowledge, historical operating information, process flows, and control loop relationships.

The continuous monitored data are processed according to the procedure described in Section 3.1. Specifically, the monitored variables are discretized into binary alarm-state sequences according to the corresponding alarm thresholds, and a sliding-window operation is applied to capture temporal variations. After data cleaning and format standardization, the resulting data are used as inputs for the proposed IF-MMHC framework.

The proposed method first applies Liang–Kleeman information flow to quantify directional information transfer among the monitored variables and filters weak information flow relationships using the significance threshold. The resulting relationships are then used to constrain the MMHC-based structure search, through which the final causal graph and corresponding alarm propagation paths are obtained. The experiments in this study focus on the predefined TEP fault scenarios listed in Table 2 rather than covering all possible IDVs. Eight monitored variables associated with these scenarios were selected based on their relevance to the investigated fault propagation processes, process flow relationships, and control loop interactions.

### 4.1. Tennessee Eastman (TE) Chemical Process

**Figure 2:**
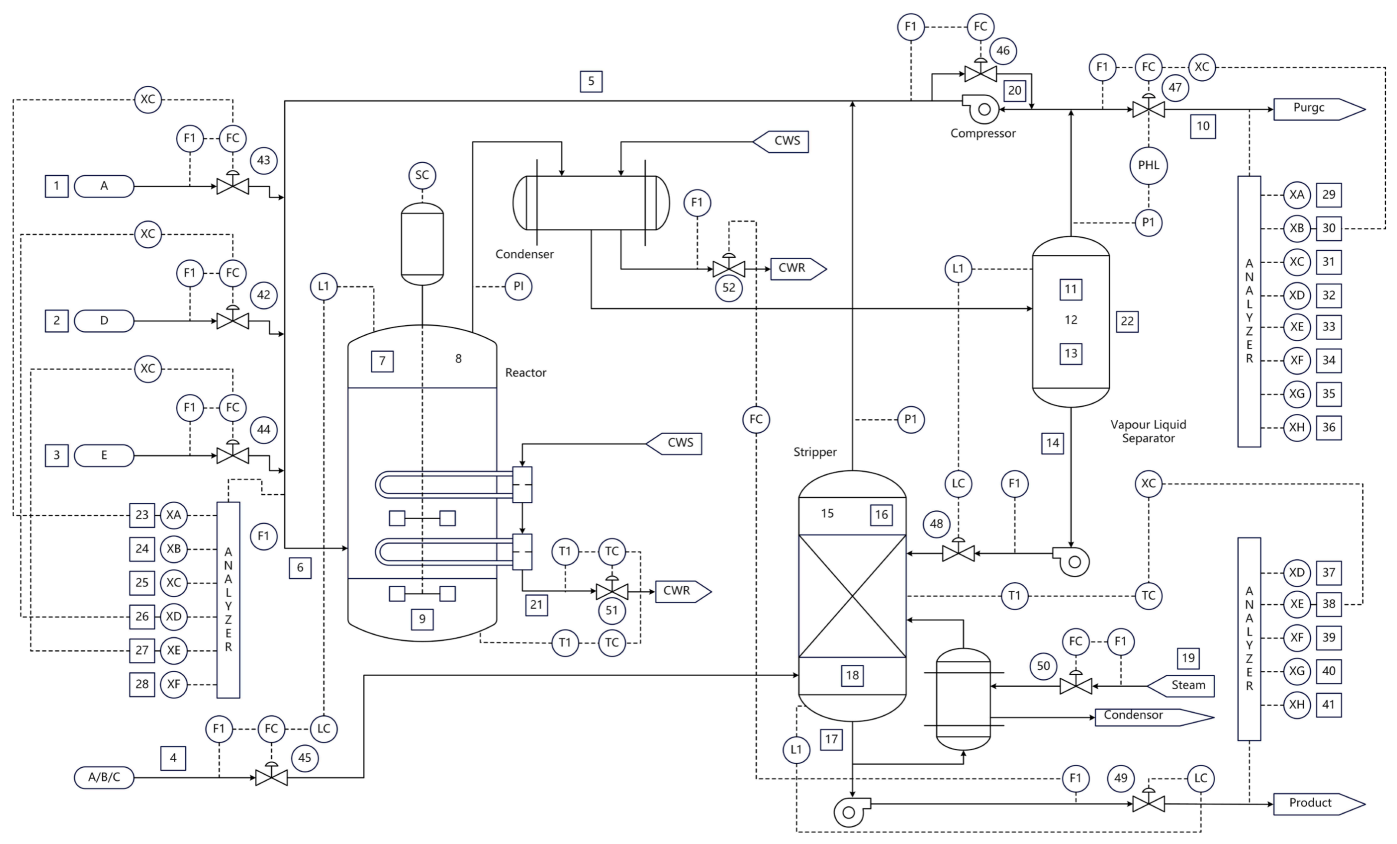
Tennessee Eastman flowchart.

The Tennessee Eastman (TE) chemical process was developed as a research experimental platform by experts Downs and Vogel from the American Chemical Company, based on real industrial chemical processes [26]. The TE process primarily consists of several operational units, including a continuous stirred-tank reactor, a condenser, a gas–liquid separator, and a reboiler. The process flow diagram is shown in Figure 2. The TE process involves four gaseous reactants, *A*, *D*, *E*, and *C,* each containing small amounts of the inert gas *B*. Under the action of a catalyst, four simultaneous chemical reactions occur in the reactor, of which two main reactions produce the liquid products G and H, described by the following reaction equations:

*A*(*g*) + *C*(*g*) + *D*(*g*) → *G*(*l*) (*Product* 1)

*A*(*g*) + *C*(*g*) + *E*(*g*) → *H*(*l*) (*Product* 1)

Additionally, two side reactions occur as follows:

*A*(*g*) + *E*(*g*) → *F*(*l*) (*By* − *product*)

3*D*(*g*) → 2*F*(*l*) (*By* − *product*)

All of these reactions are irreversible and exothermic. According to the Arrhenius equation, their reaction rates are temperature-dependent. The reaction producing product *G* has a particularly high activation energy and is highly sensitive to temperature variations. Moreover, these chemical reactions generally follow first-order kinetics with respect to the reactant concentrations, and their rate constants conform to the Arrhenius equation.

### 4.2. Simulation Experiment Setup

Prior to implementing the proposed method, representative industrial operating scenarios were constructed based on the Tennessee Eastman (TE) Process simulation platform to generate dynamic process data. Specifically, the TEP model was used to simulate a continuous 48 h operation, incorporating feed stream fault scenarios such as disturbances in the A/C feed ratio, a constant B component, and abnormal opening/closing of the reactor cooling water valve. In this study, the process data were sampled at a 3 min interval (20 samples/h) for the causal analysis experiments. This temporal resolution is also consistent with that adopted in previous Tennessee Eastman Process studies for plant-wide process monitoring and fault tracing [27]. These faults primarily affect the reactor unit; however, due to the complex coupling among process variables and cross-unit causal relationships, they often induce cascading alarms triggered by non-root-cause variables, substantially increasing the difficulty of alarm identification and imposing higher requirements on alarm configuration strategies.

To accurately identify redundant alarms and extract critical alarm pathways, variables closely related to the reactor unit and feed system were selected as monitored variables, and their dynamic behavior was analyzed to screen key alarm variables. The specific fault settings used in the experiments are listed in Table 2, and the definitions and descriptions of monitored variables are provided in Table 3.

The experiments in this study focus on the predefined TEP fault scenarios listed in Table 2 rather than covering all possible IDVs. Eight monitored variables associated with these scenarios were selected based on their relevance to the investigated fault propagation processes, process flow relationships, and control loop interactions.

For the evaluation of AC@k and Avg@k, the reference root causes are determined from the predefined fault configurations of the TEP simulation scenarios. The fault-source variables specified by each fault configuration are treated as the reference root-cause variables. These reference variables are defined independently of the proposed IF-MMHC results and are not inferred from the causal graph produced by the proposed method.

The 3 min sampling interval corresponds to 20 samples per hour. Accordingly, a window size of 10–15 samples corresponds to 30–45 min of observations used for local estimation. The time-lag parameter was set to *τ* = 2 in the main experiments, corresponding to two sampling intervals (6 min). These parameters describe the temporal configuration of the causal estimation procedure and should not be directly equated with the reported responsiveness. The reported responsiveness of 20–30 min refers to the elapsed time from fault propagation to identification of the corresponding reference root-cause variables under the investigated scenarios.

To distinguish high-threshold and low-threshold alarms from the monitored variable data, the 3σ criterion was employed to determine the upper and lower thresholds [28]. These thresholds are distinct from the 0.5 threshold used during binary-state restoration after moving-average processing.

$$L_{a}=\{\begin{array}{cc} 1, & V_{P}\left(t\right)\leq \mu -3\sigma \;\\ 0, & V_{p}(t)>\mu -3\sigma \end{array}$$

$$H_{a}=\{\begin{array}{cc} 1, & V_{P}\left(t\right)\geq \mu +3\sigma \;\\ 0, & V_{p}(t)<\mu +3\sigma \end{array}$$

Here, *μ* and *σ* denote the mean and standard deviation of the monitored variable under normal operating conditions, respectively, and *V_P_*(*t*) represents the process data of the variable at time t. *L_a_* and *H_a_* represent the low and high thresholds of the variable, respectively. The high and low thresholds of each monitored variable are shown in Table 4.

The resulting binary alarm sequences were subsequently processed using the moving-average procedure described in Section 3.1, with a window length of 10 samples and a step size of 2. The averaged values were re-binarized using the threshold of 0.5 before causal analysis. Otherwise, The corresponding root-cause variables for each fault scenario are summarized in Table 5.

## 5. Results and Discussion

### 5.1. Experimental Results

**Figure 3:**
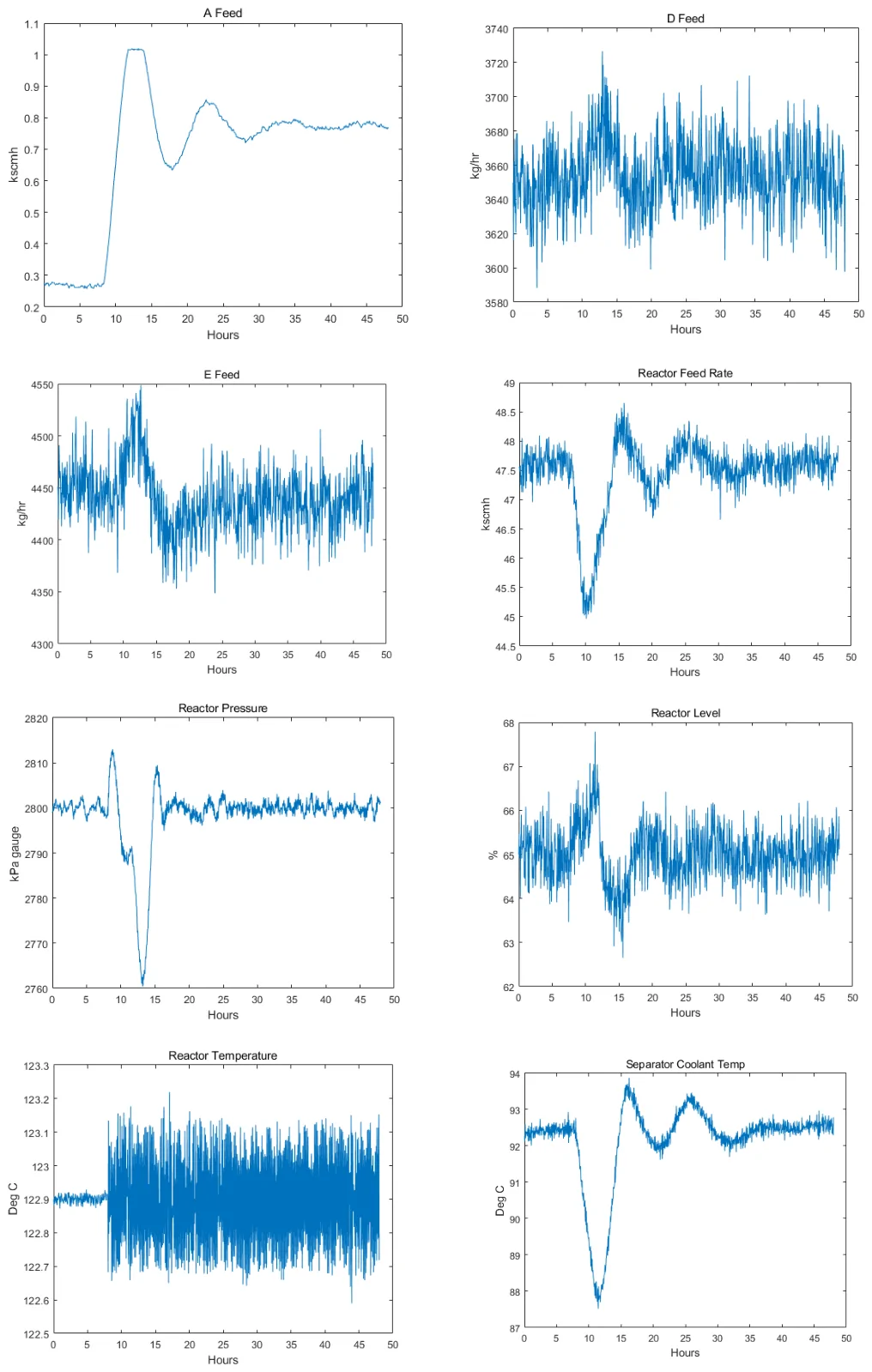
Experimental results.

To gain a deeper understanding of the dynamic impact of fault disturbances on key monitored variables, Figure 3 illustrates the response trends of the major variables during the TE simulation. At the 8th hour, the system was simultaneously subjected to two disturbances: an A/C feed ratio imbalance and sticking of the cooling water valve. The A/C feed ratio disturbance caused the reactant ratio to deviate from its normal operating condition. In particular, the deficiency of the limiting component C reduced the main reaction rate and consequently decreased the heat-release intensity, contributing to a decrease in reactor temperature (x09) and activation of the low-temperature alarm (x09_low). Meanwhile, cooling water valve sticking introduced nonlinear fluctuations into the cooling system. Because the valve could not regulate the cooling water flow smoothly, the control action required to overcome static friction caused abrupt changes in valve opening, resulting in limit-cycle oscillations of the cooling water flow. This oscillatory behavior was directly reflected in the reactor temperature (x09), which exhibited pronounced high-frequency fluctuations after the 8th hour.

The temperature disturbance also affected the thermal load of the downstream cooling system. As the heat transfer conditions changed, the cooling water outlet temperature (x21) exhibited pronounced fluctuations and subsequently triggered the low-temperature alarm (x21_low). Meanwhile, to respond to the changed process conditions and maintain the desired material and thermal balance, the control system increased the A feed (x01). Due to control lag and the nonlinear superposition of the two disturbances, the A feed subsequently overshot, triggering the high-limit A feed alarm (x01_high). The resulting imbalance in the feed composition further affected the overall material balance and caused a decrease in the total feed rate (x06), which, after a certain time delay, triggered the low-limit feed rate alarm (x06_low).

The combined effects of the feed imbalance, reactor temperature fluctuations, and subsequent control actions further affected the reactor pressure (x07) and reactor level (x08). The resulting changes in reaction conditions and gas-phase behavior caused the reactor pressure to increase and contributed to the abnormal variation in reactor level. As the control system responded to these deviations, the reactor feed rate was substantially reduced, while the downstream variables continued to exhibit dynamic fluctuations. In particular, the cooling water outlet temperature (x21) subsequently increased as the thermal conditions of the process evolved, leading to the activation of the high-limit x21 alarm (x21_high). The reactor temperature (x09) also exhibited a corresponding upward response, eventually triggering the high-limit reactor-temperature alarm (x09_high).

As the total feed rate continued to decrease and the reaction approached a low-conversion state, the reactor pressure subsequently decreased, eventually triggering the low-limit x07 alarm (x07_low). To compensate for the resulting material imbalance, the control system adjusted the E feed (x03) and the recycle flow. The delayed control response caused further fluctuations in the downstream process variables and eventually contributed to an abnormal increase in reactor level, triggering the high-limit x08 alarm (x08_high).

Overall, the two simultaneous disturbances produced a clear temporal propagation pattern: initial disturbance responses in the actuator and process variables (high-frequency fluctuations in reactor temperature x09 and a step increase in A feed x01) → changes in key reactor states (variations in reactor pressure x07 and level x08) → intervention of the feed-control loop (a substantial reduction in reactor feed rate x06) → downstream thermal load fluctuations and compensatory feed adjustments (fluctuations in separator cooling water temperature x21 and D/E feeds x02/x03). This chain response reflects the dynamic propagation of process disturbances and the resulting alarm activation sequence, providing the basis for the subsequent causal modeling and alarm propagation analysis.

### 5.2. Parameter Sensitivity Analysis

**Figure 4:**
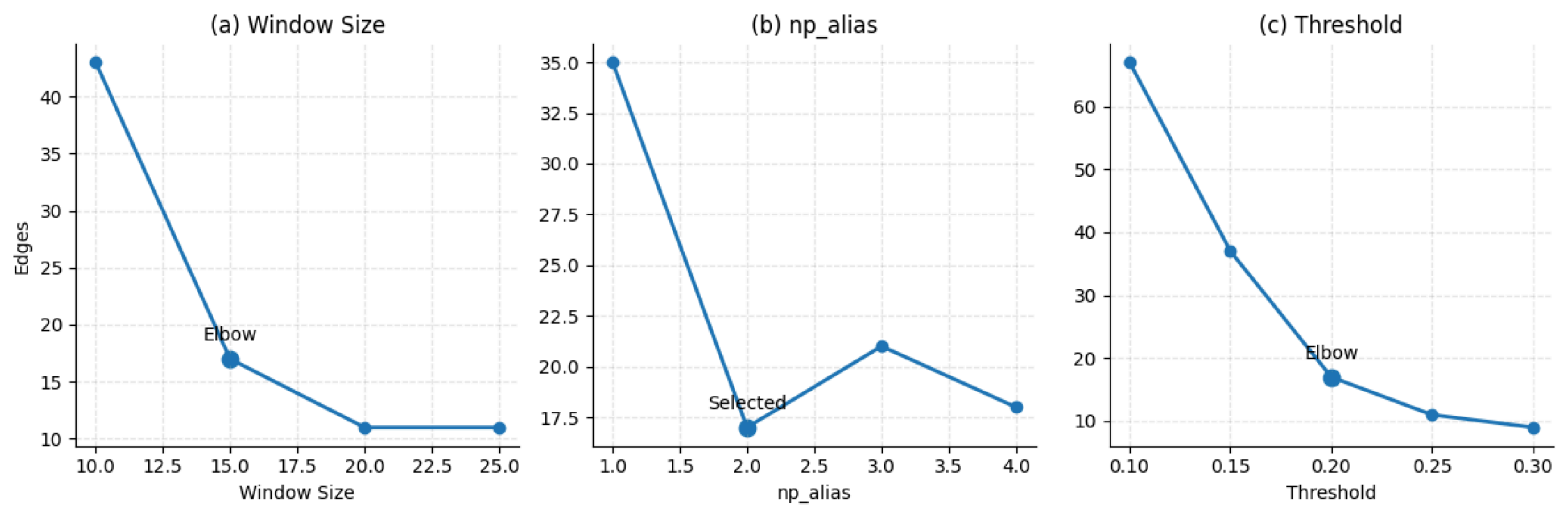
Sensitivity analysis of the window size, time lag parameter (np_alias), and significance threshold.

This section further conducts a sensitivity analysis of the key parameters in the model, including the window size, the time lag parameter (np_alias), and the significance threshold; the results are illustrated in Figure 4. Different parameter selection criteria are adopted according to the characteristics of each parameter. For the window size and significance threshold, the elbow method is used to identify the point at which the variation in the number of network edges changes from relatively rapid to relatively gradual. In contrast, because np_alias does not exhibit a monotonic trend, its value is selected by comparing the resulting network structures and the ability to characterize temporal dependencies.

First, regarding the window size parameter, when it increases from 10 to 15, the number of network edges decreases significantly, from 43 to 17, after which the decrease becomes much smaller. This indicates a clear elbow characteristic, and therefore, a window size of 15 is selected in this study.

For the significance threshold parameter, the number of network edges gradually decreases as the threshold increases. Around a threshold of 0.2, the decrease in the number of edges changes from relatively rapid to relatively gradual, indicating an elbow point. Therefore, a significance threshold of 0.2 is selected as the final configuration.

For the time lag parameter (np_alias), four candidate values, namely 1, 2, 3, and 4, are evaluated. As shown in Figure 4b, the number of network edges decreases sharply from 35 at np_alias = 1 to 17 at np_alias = 2, then increases to 21 at np_alias = 3 and decreases slightly to 18 at np_alias = 4. Thus, no monotonic trend or clear elbow point is observed, and the elbow method is not applied to this parameter. Instead, np_alias = 2 is selected by jointly considering the temporal dependency captured by the model and the resulting network structure. A value of 2 provides a relatively compact network while retaining the temporal information required for characterizing the propagation relationships. Therefore, np_alias = 2 is adopted in the subsequent experiments.

### 5.3. Results Analysis

**Figure 5:**
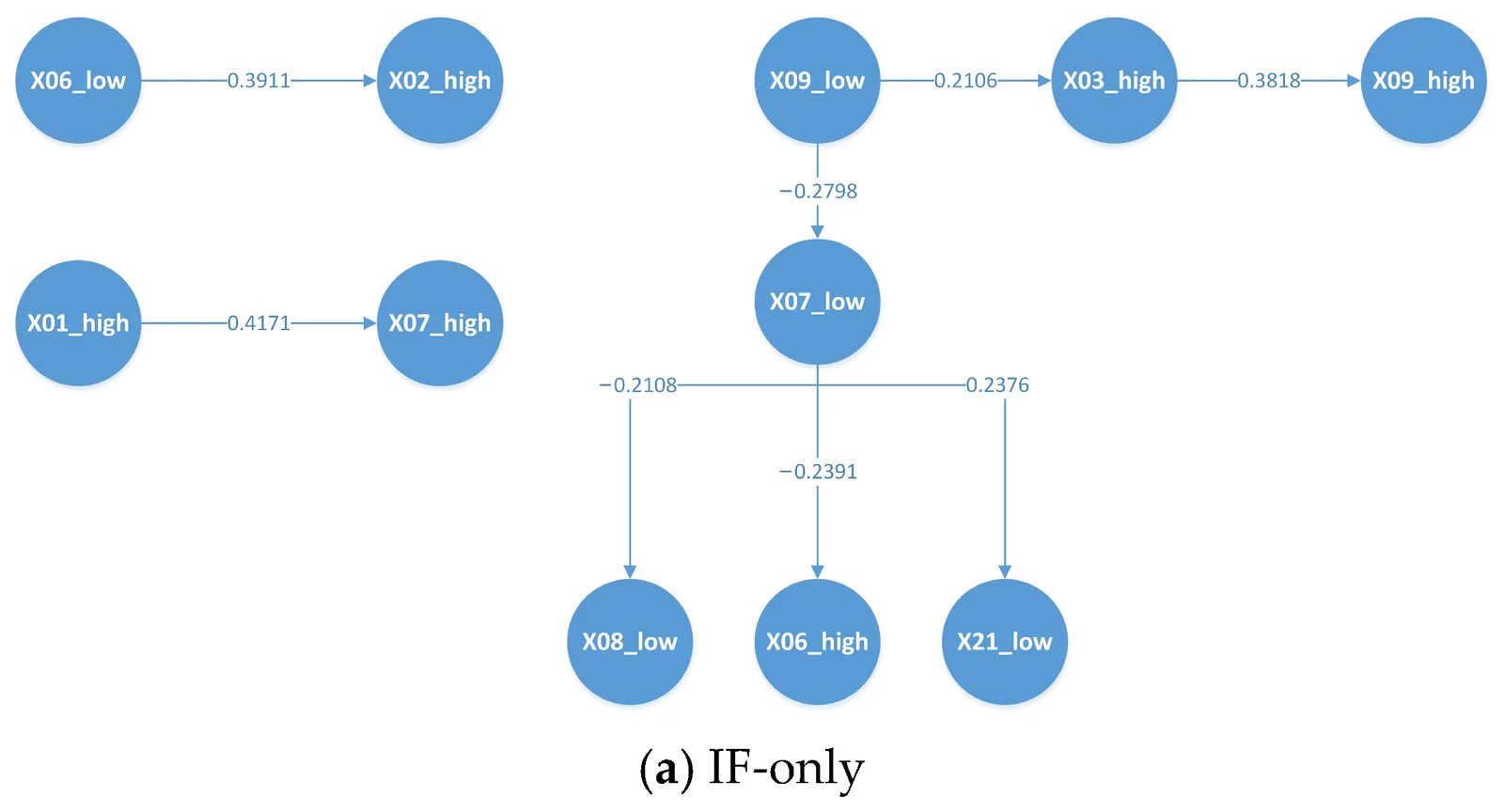

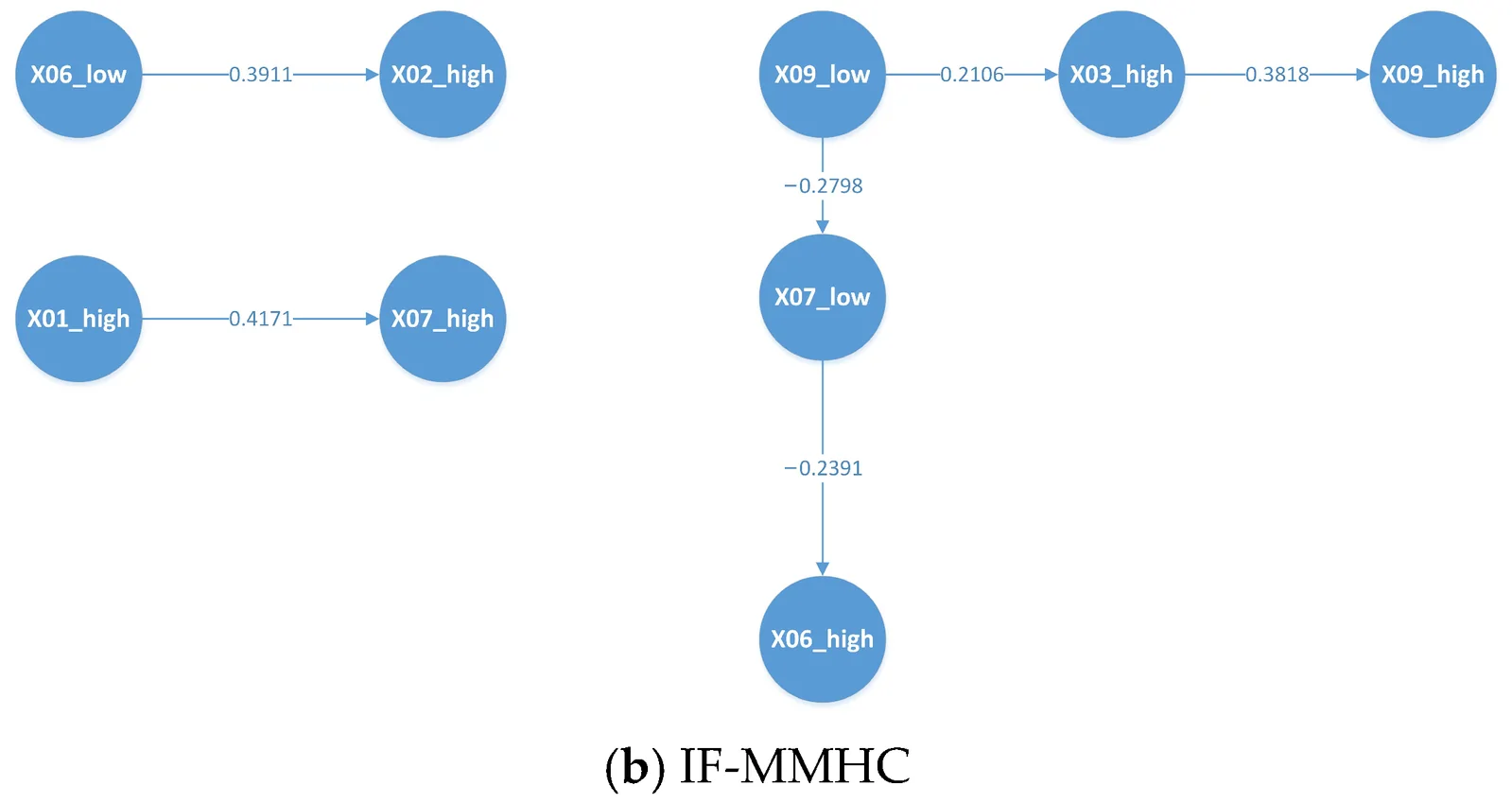
Comparison of the causal graphs obtained by (**a**) LKIF-only and (**b**) IF-MMHC.

To examine the contribution of the MMHC refinement stage, the causal graph obtained after LKIF-based information flow estimation and threshold filtering is compared with the final graph obtained by IF-MMHC, as shown in Figure 5. The ablation analysis compares LKIF-only and the full IF-MMHC framework. A direct MMHC-only ablation is not included because the MMHC stage in the proposed framework operates within the CPC candidate search space generated by LKIF. Removing LKIF would require an independently defined candidate generation procedure, resulting in a separate MMHC baseline rather than a direct component-wise ablation of the proposed framework.

The LKIF-only graph provides the initial directional causal relationships, whereas the subsequent MMHC stage further refines the graph structure under the directional constraints provided by LKIF.

As shown in Figure 5b, the estimated information flow relationships capture several physically meaningful material, energy, and control response dependencies in the TEP. For example, the path from x01_high to x07_high indicates that an abnormal increase in A-feed flow is associated with a subsequent increase in reactor pressure. This relationship is consistent with the material and thermodynamic dependencies of the reactor, where changes in feed composition and flow affect the amount of material entering the reactor and consequently its pressure state.

Similarly, the path from x09_low to x07_low reflects the dynamic coupling between reactor temperature and pressure. A decrease in reactor temperature is accompanied by a subsequent decrease in reactor pressure, indicating the propagation of the disturbance from the reactor thermal state to its pressure state.

The information flow paths also reveal the responses of the closed-loop control system. For example, the paths from x06_low to x02_high and from x07_low to x06_high indicate compensatory control actions following abnormal changes in the total feed rate or reactor pressure. These relationships reflect the response of the control system to process disturbances, in which changes in one process variable induce adjustments in other manipulated variables to maintain stable operation.

In addition, the paths from x09_low to x03_high and from x03_high to x09_high form a closed-loop interaction between reactor temperature and E-feed flow. A decrease in reactor temperature is followed by an increase in E feed, while the subsequent change in E feed is associated with an increase in reactor temperature. This relationship reflects the interaction between process dynamics and control actions and provides an example of how feedback effects can propagate through the alarm network.

It should be noted that the sign and magnitude of the information flow weight have different meanings. The sign of the information flow value represents the direction of the causal effect, whereas its absolute magnitude reflects the strength of the estimated information flow. Therefore, the negative weight associated with the x01_high → x07_high relationship indicates a negative causal effect between the two variables, rather than a “rigid constraint” imposed by mass conservation. The magnitude of the information flow value reflects the strength of this causal relationship. The physical interpretations above therefore provide process-level context for understanding the estimated information flow relationships and help assess their consistency with the underlying process dynamics.

### 5.4. Performance Metrics

To evaluate the practical effectiveness of the proposed framework for alarm root-cause identification, IF-MMHC was compared with representative methods based on different causal or dependency analysis principles, including TE–K2 [2], GC [29], Pearson correlation [30], Apriori association rule mining [31], and DBN [17], as summarized in Table 6.

All baseline methods were evaluated using the same preprocessed and discretized alarm dataset described above, under the same fault scenarios and evaluation settings as IF-MMHC. Where applicable, the corresponding method-specific threshold or parameter was calibrated separately using the elbow point procedure described in Section 5.2, because the raw statistics produced by different methods are not directly comparable on a common numerical scale. This design ensures that the comparison focuses on the root-cause identification performance of each method rather than differences arising from the scale of their respective statistical measures.

These methods do not share an identical structure-learning architecture. In particular, IF-MMHC combines directional information flow analysis with MMHC-based structure refinement, whereas TE–K2 combines transfer entropy with K2-based Bayesian network learning. GC, Pearson correlation, and Apriori provide alternative temporal–causal, correlation-based, and association rule-based analyses, respectively, while DBN performs dynamic Bayesian network structure learning. Therefore, the comparison focuses on their empirical root-cause identification performance under the same TEP fault scenarios rather than treating these methods as structurally equivalent algorithms.

Based on the results in Table 6, the six methods—IF-MMHC, TE–K2, GC, Pearson, Apriori, and DBN—exhibit different characteristics in terms of responsiveness and fault-source ranking performance. AC@k and Avg@k evaluate the ranking performance of predefined fault-source variables. Therefore, these metrics reflect different aspects of method performance and should be interpreted jointly rather than as a single overall measure.

For IF-MMHC, both AC@k and Avg@k reach 100%. According to the definitions of these metrics, this means that the predefined fault-source variables are consistently included in the corresponding top-k ranked results under the evaluated TEP fault scenarios. Therefore, the perfect AC@k and Avg@k values should be interpreted as evidence of successful fault-source ranking under the tested scenarios, rather than as direct evidence that all causal directions or propagation paths are correctly reconstructed.

An important point is that Pearson correlation also achieves 100% for both AC@k and Avg@k. This result does not indicate that Pearson correlation can identify directional causal relationships. Pearson correlation is a symmetric measure of statistical association and therefore does not distinguish causes from effects. Therefore, the 100% AC@k and Avg@k achieved by Pearson should be interpreted as successful root-cause ranking under the tested scenarios rather than as evidence of causal identification or directional causal structure reconstruction. In contrast, IF-MMHC additionally provides directional information flow relationships and a refined causal graph structure for analyzing alarm propagation. In the present evaluation, the Pearson-based results are ranked according to the strength of statistical association, while AC@k and Avg@k only evaluate whether the predefined fault-source variables appear among the highly ranked variables. Consequently, Pearson correlation can obtain perfect AC@k and Avg@k when the fault-source variables exhibit sufficiently strong statistical associations with the observed alarm variables, even though the resulting associations do not provide causal direction.

In terms of responsiveness, IF-MMHC requires approximately 20–30 min under the experimental settings, while DBN requires 30–45 min, TE–K2 and GC require approximately 40 min–1 h, Pearson requires approximately 40 min–1 h, and Apriori requires more than 1 h. These results indicate that the evaluated IF-MMHC implementation provides relatively short response time under the tested TEP scenarios. However, the reported responsiveness depends on the experimental configuration, implementation, and dataset, and should therefore not be interpreted as a universal computational advantage across all industrial applications.

Overall, the comparison shows that the evaluated methods exhibit different characteristics in terms of fault-source ranking and responsiveness, while methods that construct causal structures additionally differ in the directional information provided by their resulting graphs. Under the tested TEP scenarios, IF-MMHC achieves 100% AC@k and Avg@k together with a responsiveness of 20–30 min. These results indicate that the proposed framework successfully ranks the predefined fault-source variables while providing a directional causal structure for analyzing alarm propagation in the evaluated cases. The perfect ranking scores are therefore interpreted together with the directional nature of the underlying causal model rather than as a standalone measure of complete causal reconstruction.

## 6. Conclusions

This paper addresses spurious causal relationships in “alarm-triggered alarm” phenomena induced by control–structure coupling in industrial systems and proposes a causal graph modeling method based on Liang’s information flow and the Max-Min Hill-Climbing algorithm (IF-MMHC). The proposed framework uses Liang’s information flow to characterize directional information transfer among process variables and integrates MMHC-based constraint checking and score-based structure learning to refine the causal graph.

Experiments on the Tennessee Eastman Process show that the constructed causal graph corresponds to several known process relationships and observed dynamic behaviors in the tested scenarios. The comparative results show that the evaluated methods exhibit different characteristics in terms of responsiveness and fault-source ranking. Under the tested alarm-triggered alarm scenarios, the proposed IF-MMHC framework identifies the predefined fault-source variables while providing a directional causal structure for analyzing alarm propagation.

The main theoretical contribution of this work is the integration of directional information flow analysis with constrained and score-based causal structure learning, which provides a framework for filtering weak information flow relationships and refining redundant causal edges. From a practical perspective, the proposed method provides a data-driven approach for identifying key alarm-source variables and analyzing alarm propagation relationships, which can support the interpretation and simplification of causal structures in industrial alarm analysis.

This study should be regarded as a methodological validation based on TEP simulation rather than a field-deployment validation. The current experiments do not include real industrial sensor and alarm event data, multiple sampling frequencies, noisy alarm labels, or switching operating conditions. In addition, alarm thresholds, sampling intervals, and closed-loop control may affect the estimated information flow relationships and causal directions. Therefore, further validation using real industrial monitoring data under diverse operating conditions is required to assess the generalizability and field applicability of the proposed framework.

Future research will investigate the extension of the proposed framework to heterogeneous industrial monitoring scenarios that combine process sensor measurements with equipment-related information and other operational data. Such extensions will focus on causal prediction and dynamic alarm threshold optimization using multimodal industrial data.

## Figures and Tables

**Table 1:** Sliding average window settings.

Parameter	Setting
Moving-average length	10 samples
Value > 0.5	1
Value < 0.5	0
Time step	2

**Table 2:** Simulation parameters.

Parameter	Setting
Total simulation duration	48 h
Fault introduction time	8th hour
Disturbance type	(IDV 1) Change in A/C feed ratio, B component constant; (IDV 14) Reactor cooling water valve abnormal
Sampling frequency	20 samples per hour

**Table 3:** Description of monitored variables.

Process Variable	Description
x_01_	A feed rate
x_02_	D feed rate
x_03_	E feed rate
x_06_	Reactor feed flow rate
x_07_	Reactor pressure
x_08_	Reactor level
x_09_	Reactor temperature
x_21_	Reactor cooling water

**Table 4:** Monitored variable high–low threshold.

Process Variable	Low Threshold	High Threshold
x_01_	0.249	0.284
x_02_	3597.394	3711.109
x_03_	4371.271	4514.069
x_06_	46.954	48.235
x_07_	2796.971	2803.033
x_08_	63.494	66.505
x_09_	122.867	122.932
x_21_	102.409	102.527

**Table 5:** Fault scenarios and reference root causes.

Fault Scenario	Fault Description	Reference Root Cause
IDV (1)	Change in A/C feed ratio,	x_06_
	B component constant;	
IDV (14)	Reactor cooling water valve	x_09_
	abnormal	

**Table 6:** Comparison of root-cause identification performance.

	IF-MMHC	TE-K2	GC	Pearson	Apriori	DBN
Responsiveness	20–30 min	40 min–1 h	40 min–1 h	40 min–1 h	>1 h	30–45 min
AC@k	100%	50%	100%	100%	50%	80%
Avg@k	100%	60%	70%	100%	60%	50%

Note: IF-MMHC and TE-K2 are hybrid approaches that combine a causal/dependency measure with a subsequent structure-learning procedure, whereas GC, Pearson correlation, Apriori, and DBN represent alternative temporal–causal, correlation-based, association rule-based, and dynamic Bayesian approaches, respectively. All methods were evaluated under the same predefined TEP fault scenarios and root-cause evaluation criteria.

## Data Availability

The data presented in this study are available from the corresponding author upon reasonable request.

## Abbreviations

The following abbreviations are used in this manuscript:

IF	Liang–Kleeman information flow
MMHC	Max-Min-Hill-Climbing (IF-MMHC)

## Appendix A

### Appendix A.1. Continuous-Time Formulation of Liang–Kleeman Information Flow

The Liang–Kleeman information flow theory, proposed by Liang X. S. and Richard Kleeman, is designed to quantify the deterministic causal information transfer among variables in dynamical systems. Through rigorous mathematical derivation, this framework characterizes the intrinsic dynamical dependencies between system variables and enables the effective identification of causal interaction strength. By decomposing the evolution of marginal entropy, the theory distinguishes endogenous variations arising from a variable’s own dynamics from exogenous influences induced by information flow from other variables. This decomposition provides a theoretical basis for separating information contributions associated with a variable’s intrinsic dynamics from those arising from cross-variable interactions, thereby reducing spurious causal interpretations associated with temporal dependence and autocorrelation.

First, consider a two-dimensional system:

(A1) $$\frac{dx_{1}}{\mathrm{dt}}=F_{1}(x_{1},x_{2},t)$$

(A2) $$\frac{dx_{2}}{\mathrm{dt}}=F_{2}(x_{1},x_{2},t)$$

This represents the minimal-dimensional system that permits information flow. Without loss of generality, we examine only the transfer from *X*_2_ variable to *X*_1_. Under the vector field, $F={\left(F_{1},{\;F}_{2}\right)}^{T}$ evolves over time; correspondingly, its joint probability density *ρ*(*x*) evolves according to a Liouville equation:

(A3) $$\frac{\partial \rho }{\partial t}\;+\frac{\partial }{\partial x_{1}}(F_{1}\rho )\;+\frac{\partial }{\partial x_{2}}(F_{2}\rho )=0\;$$

What is important here is the evolution of *H*_1_, i.e., the marginal entropy of *x*_1_. Integrating (A3) over *x*_2_ on R, we obtain the following:

(A4) $$\frac{\partial \rho }{\partial t}\;+\frac{\partial }{\partial x_{1}}{\int \left(F_{1}\rho \right)}_{R}\;dx_{2}=0$$

Due to the assumption of compact support for *ρ*, multiplying (A4) by −(1 + logρ_1_), and integrating over ***R***, yields the following trend for the entropy of *H*_1_:

(A5) $$\frac{dH_{1}}{\mathrm{dt}}={\int \left[\log\rho_{1}\frac{\partial (F_{1}\rho )}{\partial x_{1}}\right]dx_{1}dx_{2}}_{R^{2}}=-E(\frac{F_{1}}{\rho_{1}}\frac{\partial \rho_{1}}{\partial x_{1}})$$

As the system drives the state forward, the marginal entropy of x_1_ is contributed by two distinct sources: one from *x*_1_ itself, and the other from *x*_2_. The latter is supplied by the information flow/transfer mechanism. According to its underlying mechanism, the increase in marginal entropy can be decomposed as follows:

(A6) $$\frac{dH_{1}}{\mathrm{dt}}=\frac{dH_{1}^{*}}{\mathrm{dt}}\;+\;T_{2\to 1}$$

Liang and Kleeman argue that only the intrinsic dynamics of *x*_1_ should contribute to the rate of change in its marginal entropy:

(A7) $$\frac{dH_{1}^{*}}{\mathrm{dt}}=\int {\rho \frac{\partial F_{1}}{\partial x_{1}}}_{R^{2}}dx_{1}dx_{2}$$

Thus, the rate of information flowing from *x*_2_ to *x*_1_ is as follows:

(A8) $$T_{2\to 1}=-\int \rho_{2|1}(x_{2}|x_{1})\frac{\partial F_{1}\rho_{1}}{\partial x_{1}}dx_{1}dx_{2}$$

Specifically, the information flow rate from variable *x*_2_ to variable *x*_1_ can be expressed as follows:

(A9) $$T_{2\to 1}=-E\left[\frac{1}{\rho_{1}}\;\frac{\partial \left(F_{1}\rho_{1}\right)}{\partial X_{1}}\right]+\;\frac{1}{2}E[\frac{1}{\rho_{1}}\;\frac{\partial^{2}(b_{11}^{2}+b_{12}^{2})\rho_{1}}{\partial x_{1}^{2}}]$$

where *ρ*_1_ is the marginal probability density of *X*_1_*, B* = (*b_ij_*) is the diffusion coefficient matrix, and E denotes expectation with respect to *ρ*(*X*_1_,*X*_2_).

Each term in this formula is derived from the mathematical properties of the system dynamics, without introducing any empirical parameters or subjective assumptions. Therefore, the computation of information flow is entirely based on objective relationships between variables.

When the evolution of X_2_ is not influenced by *X*_1_, the information flow from *X*_2_ to *X*_1_ is zero, i.e., *T*_2→1_ = 0.

In real industrial alarm systems, there often exist implicit high-dimensional coupling structures. If there are unobserved common driving variables *X*_3_, *X*_4_, …, *X_n_*, then the entropy rate of a variable may include contributions from these latent factors. In such cases, if analysis is conducted only based on low-dimensional modeling or simple conditional mutual information frameworks, it may lead to spurious causality induced by statistical correlations from common drivers; spurious information flow introduced by autocorrelation structures; and distorted information decomposition due to insufficient conditioning sets.

Therefore, a more rigorous approach is to derive the computation formulas for $\frac{dH_{1}}{\mathrm{dt}}$ and $\frac{dH_{12}}{\mathrm{dt}}$, expressing *T*_2→1_ in a strict mathematical form. By decomposing the drift and diffusion terms in the system’s differential equations, this method clearly distinguishes contributions from a variable’s own dynamics and contributions from cross-variable coupling terms. Thus, information flow *T*_2→1_ is determined solely by structural interaction terms.

In other words, the computation of the information flow term analytically separates the contribution associated with the intrinsic dynamics of the receiving variable from the contribution attributable to cross-variable information transfer. Therefore, temporal dependence associated with autoregressive or intrinsic dynamical effects is not directly interpreted as information flow from another variable. This theoretical decomposition provides a basis for reducing spurious causal interpretations arising from autocorrelation and background dynamical effects.

This theoretical property ensures that the Liang–Kleeman information flow (LKIF) method can effectively suppress spurious causality in complex industrial alarm systems and achieve precise characterization of information flow based on system dynamics.

Based on the following system:

*dx* = *F*(*t*:*x*)*dt* + *B*(*t*:*x*)*dw* (A10)

where *x* and *F* are N-dimensional vectors, *B* is an ***n × m*** matrix, and *w* is an m-dimensional standard Wiener process.

Let *B_i_* denote the i-th component of vector. Differentiating the above equation gives the following:

*x*(*t*+Δ*t*) = *x*(*t*) + *F*Δ*t* + *B*Δ*w* (A11)

Le*t BB^T^* ≡ *G* ≡ (*g_ij_*); the time change rate of H_i_ is as follows:

(A12) $$\frac{dH_{i}}{\mathrm{dt}}=-E[F_{i}\frac{\partial \log\rho_{i}}{\partial x_{i}}]\;-\;\frac{1}{2}E[g_{\mathrm{ii}}\frac{\partial^{2}\log\rho_{i}}{\partial x_{i}^{2}}]$$

Let *ρ_i_* be defined as $\int_{R}\rho (x)dx_{2}$:

$$\frac{dH_{\mathrm{ij}}}{\mathrm{dt}}=-E[F_{i}\frac{\partial \log\rho_{i}}{\partial x_{i}}]\;-\;\frac{1}{2}E[g_{\mathrm{ii}}\frac{\partial^{2}\log\rho_{i}}{\partial x_{i}^{2}}]\;+\;E[\frac{1}{\rho_{i}}\int_{R^{n-2}}\frac{\partial F_{i}\rho_{j}}{\partial x_{i}^{2}}dx_{1}\ldots x_{i-1}x_{i+1}\ldots x_{j-1}x_{j+1}\ldots x_{n}]\;-\;\frac{1}{2}E[\frac{1}{\rho_{i}}\int_{R^{n-2}}\frac{\partial g_{\mathrm{jj}}\rho_{j}}{\partial x_{i}^{2}}dx_{1}\ldots x_{i-1}x_{i+1}\ldots x_{j-1}x_{j+1}\ldots x_{n}]$$

Subtracting the above two expressions yields the following:

(A13) $$T_{j\to i}=\int_{R^{3}}\rho_{j|i}(x_{j}|x_{i})\frac{\partial F_{i}\rho_{j}}{\partial x_{i}}\mathrm{dx}-\frac{1}{2}\int_{R^{n}}\rho_{j|i}(x_{j}|x_{i})\frac{\partial^{2}(b^{2}\rho_{j})}{\partial x_{i}^{2}}\mathrm{dx}$$

In other words, the computation of the information flow term separates the contribution associated with the intrinsic dynamics of the receiving variable from the contribution attributable to cross-variable information transfer. Therefore, temporal dependence associated with autoregressive or intrinsic dynamical effects is not directly interpreted as information flow from another variable. This theoretical decomposition provides a basis for using the Liang–Kleeman information flow (LKIF) method to distinguish intrinsic dynamical effects from cross-variable information transfer and thereby reduce spurious causal interpretations in complex industrial alarm systems.

### Appendix A.2. Discrete-Time Mapping Formulation and the Principle of Nil Causality

The continuous-time derivation presented above provides the theoretical foundation of Liang–Kleeman information flow. The same notion has been rigorously extended to discrete-time dynamical systems (mappings). Consider an n-dimensional discrete mapping

*x*(*τ* + 1) = Φ(*x*(*τ*)), (A14)

where Φ: ℝ^n^ → ℝ^n^. As the state is advanced from time step τ to τ + 1, the associated probability density is transported by the Frobenius–Perron operator p. The information flow from component *x*_2_ to component *x*_1_ is defined by the exclusive decomposition of the marginal-entropy increment:

*T*_2→1_ = Δ*H*_1_ − Δ*H*_1/2_ (A15)

where Δ*H*_1/2_ denotes the change in the marginal entropy of *x*_1_ when the influence of *x*_2_ is instantaneously excluded (i.e., *x*_2_ is frozen as a parameter).

When Φ is invertible, the information flow admits the closed-form expression

(A16) T2→1=Elog(p2ρ)1(Φ1(x))−Elog(pρ)1(Φ1(x)),

in which p2 is the Frobenius–Perron operator of the mapping with *x*_2_ held fixed. An analogous result holds for stochastic discrete mappings.

A fundamental property of this formulation is the principle of nil causality (Theorem 3.4 of Liang [20]):

If the evolution of *x*_1_ does not depend on *x*_2_ (i.e., Φ_1_ is independent of *x*_2_), then *T*_2→1_ = 0.

This statement is a rigorously proved theorem rather than an empirical observation. Consequently, the causal interpretability of Liang–Kleeman information flow is retained for discrete-time systems, including the binary (0/1) alarm-state sequences employed in the present study.

### Appendix A.3. Practical Linear Estimator for Binary Time Series

When only observational time series are available, the information flow rate can be estimated under a linear stochastic model. Following Liang [32], the maximum-likelihood estimator takes the compact form

(A17) $${\hat{T}}_{2\to 1}=\frac{C_{11}C_{12}C_{2,d1}-C_{12}^{2}C_{1,d1}}{C_{11}^{2}C_{22}-C_{11}C_{12}^{2}},$$

where *C_ij_* denotes the sample covariance between series i and j, and *C_i_*_,*dj*_ is the sample covariance between series i and the finite-difference series of j.

In the linear case, the estimator reduces to $T_{2\to 1}=a_{12}\frac{\sigma_{12}}{\sigma_{11}}$. Consequently, when the coupling coefficient *a*_12_ = 0, the estimated information flow vanishes, thereby inheriting the principle of nil causality.

Although the original derivation of the estimator assumes real-valued time series, it depends solely on sample covariances. These covariances remain well defined for binary (0/1) sequences. Therefore, the same estimator can be applied directly to the preprocessed binary alarm-state time series used in the present study. In this work, the estimator is evaluated locally inside sliding windows to accommodate possible non-stationarity while preserving the causal interpretability guaranteed by the underlying theory.

## References

[1] Alinezhad H.S., Roohi M.H., Chen T. (2022). A review of alarm root cause analysis in process industries: Common methods, recent research status and challenges. Chem. Eng. Res. Des..

[2] de Abreu R.S., Chen T. (2021). A method for detecting causal relationships between industrial alarm variables using Transfer Entropy and K2 algorithm. J. Process Control.

[3] Luo Y., Li Y., Chen T. (2023). Adaptive online optimization of alarm thresholds using multilayer Bayesian networks and active transfer entropy. Control Eng. Pract..

[4] Song X., Liu Q., Dong M., Meng Y., Qin C., Zhao D., Yin F., Jiu J. (2022). Chemical process alarm root cause diagnosis method based on the combination of data-knowledge-driven method and time retrospective reasoning. ACS Omega.

[5] Herrera M., Benítez D., Pérez-Pérez N., Di Teodoro A., Camacho O. (2023). Hybrid controller based on numerical methods for chemical processes with a long time delay. ACS Omega.

[6] Liang X.S. (2013). The Liang-Kleeman information flow: Theory and applications. Entropy.

[7] Rao H.R.M., Zhou B., Brown K., Chen T., Shah S.L. (2024). Alarm correlation analysis with applications to industrial alarm management. Control Eng. Pract..

[8] Tsamardinos I., Brown L.E., Aliferis C.F. (2006). The max-min hill-climbing Bayesian network structure learning algorithm. Mach. Learn..

[9] Zhang X., Hu W., Al-Dabbagh A.W., Wang J. (2024). Improved similarity analysis of industrial alarm flood sequences by considering alarm correlations. J. Process Control.

[10] Yang B., Wang H.R., Li H.G., He Y.D. (2020). A novel detection of correlated alarms with delays based on improved block matching similarities. ISA Trans..

[11] Parvez M.R., Hu W., Chen T. (2023). An association rule mining approach to predict alarm events in industrial alarm floods. Control Eng. Pract..

[12] Yang S., Zhang T., Zhai Y., Wang K., Zhao G., Tu Y., Cheng L. (2023). Frequent alarm pattern mining of industrial alarm flood sequences by an improved PrefixSpan algorithm. Processes.

[13] Li D., Cheng X. (2024). A First-Out Alarm Detection Method via Association Rule Mining and Correlation Analysis. Entropy.

[14] Su J., Wang D., Zhang Y., Yang F., Zhao Y., Pang X. (2017). Capturing causality for fault diagnosis based on multi-valued alarm series using transfer entropy. Entropy.

[15] Luo Y., Gopaluni B., Xu Y., Cao L., Zhu Q.X. (2020). A novel approach to alarm causality analysis using active dynamic transfer entropy. Ind. Eng. Chem. Res..

[16] Chen R., Kang J.L., Wang J.G., Yao Y., Liu L.L., Xie Z.T. (2025). Granger causality analysis using error correction model for root cause diagnosis in non-stationary industrial processes. J. Taiwan Inst. Chem. Eng..

[17] Yuan L., Han G., Dong P. (2026). Improved Bayesian network with graph attention and prior algorithm for aircraft engine fault root cause analysis. Sci. Rep..

[18] Wang H., Liu R., Ding S.X., Hu Q., Li Z., Zhou H. (2024). Causal-trivial attention graph neural network for fault diagnosis of complex industrial processes. IEEE Trans. Ind. Inform..

[19] Peng K., Chen J., Yang H., Qin X. (2024). Knowledge-data-driven process monitoring based on temporal knowledge graphs and supervised contrastive learning for complex industrial processes. J. Process Control.

[20] Yang F., Cui Y., Wu F., Zhang R. (2021). Fault monitoring of chemical process based on sliding window wavelet DenoisingGLPP. Processes.

[21] Liang X.S. (2016). Information flow and causality as rigorous notions ab initio. Phys. Rev. E.

[22] Barroso F., Gomes D., Baxter G.J. (2024). Inference of Causal Networks using a Topological Threshold. IEEE Access.

[23] Liu X., Gao X., Wang Z., Ru X., Zhang Q. (2023). A metaheuristic causal discovery method in directed acyclic graphs space. Knowl.-Based Syst..

[24] Song W., Gong H., Wang Q., Zhang L., Qiu L., Hu X., Han H., Li Y., Li R., Li Y. (2022). Using Bayesian networks with Max-Min Hill-Climbing algorithm to detect factors related to multimorbidity. Front. Cardiovasc. Med..

[25] Pham L., Ha H., Zhang H. (2024). Root cause analysis for microservice system based on causal inference: How far are we?. Proceedings of the 39th IEEE/ACM International Conference on Automated Software Engineering (ASE).

[26] Downs J.J., Vogel E.F. (1993). A plant-wide industrial process control problem. Comput. Chem. Eng..

[27] Chen X., Yang Y., Wang J. (2024). Plant-Wide Processes Monitoring and Fault Tracing Based on Causal Graphical Model. IET Control. Theory Appl..

[28] Reisch R.T., Hauser T., Lutz B., Tsakpinis A., Winter D., Kamps T., Knoll A. (2022). Context awareness in process monitoring of additive manufacturing using a digital twin. Int. J. Adv. Manuf. Technol..

[29] Lv F., Yang B., Yu S., Zou S., Wang X., Zhao J., Wen C. (2025). A unified model integrating Granger causality-based causal discovery and fault diagnosis in chemical processes. Comput. Chem. Eng..

[30] Li Z., Yang Y., Li L., Wang D. (2023). A weighted Pearson correlation coefficient based multi-fault comprehensive diagnosis for battery circuits. J. Energy Storage.

[31] Zhang X., Tang Y., Liu Q., Liu G., Ning X., Chen J. (2021). A fault analysis method based on association rule mining for distribution terminal unit. Appl. Sci..

[32] Liang X.S. (2014). Unraveling the Cause-Effect Relation between Time Series. Phys. Rev. E.

